# The prevalence of clinically diagnosed ankylosing spondylitis and its clinical manifestations: a nationwide register study

**DOI:** 10.1186/s13075-015-0627-0

**Published:** 2015-05-09

**Authors:** Sofia Exarchou, Ulf Lindström, Johan Askling, Jonas K Eriksson, Helena Forsblad-d’Elia, Martin Neovius, Carl Turesson, Lars Erik Kristensen, Lennart TH Jacobsson

**Affiliations:** Rheumatology, Department of Clinical Sciences, Malmö, Lund University, Inga Marie Nilssons gata 32, 205 02 Malmö, Sweden; Department of Rheumatology and Inflammation Research, Sahlgrenska Academy at University of Gothenburg, Gothenburg, Sweden; Clinical Epidemiology Unit & Rheumatology Unit, Department of Medicine Solna, Karolinska Institute, Stockholm, Sweden; Clinical Epidemiology Unit, Department of Medicine Solna, Karolinska Institute, Stockholm, Sweden; The Parker Institute, Department of Rheumatology, Bispebjerg and Frederiksberg Hospital, the Capital Region of Copenhagen, Copenhagen, Denmark

## Abstract

**Introduction:**

Prevalence estimates of ankylosing spondylitis vary considerably, and there are few nationwide estimates. The present study aimed to describe the national prevalence of clinically diagnosed ankylosing spondylitis in Sweden, stratified according to age, sex, geographical, and socio-economic factors, and according to subgroups with ankylosing spondylitis-related clinical manifestations and pharmacological treatment.

**Methods:**

All individuals diagnosed with ankylosing spondylitis according to the World Health Organization International Classification of Disease codes, between 1967 and 2009, were identified from the National Patient Register. Data regarding disease manifestations, patient demographics, level of education, pharmacological treatment, and geographical region were retrieved from the National Patient Register and other national registers.

**Results:**

A total of 11,030 cases with an ankylosing spondylitis diagnosis (alive, living in Sweden, and 16 to 64 years old in December 2009) were identified in the National Patient Register, giving a point prevalence of 0.18% in 2009. The prevalence was higher in northern Sweden, and lower in those with a higher level of education. Men had a higher prevalence of ankylosing spondylitis (0.23% versus 0.14%, *P* < 0.001), a higher frequency of anterior uveitis (25.5% versus 20.0%, *P* < 0.001) and were more likely to receive tumor necrosis factor inhibitors than women (15.6% versus 11.8% in 2009, *P* < 0.001). Women were more likely than men to have peripheral arthritis (21.7% versus 15.3%, *P* < 0.001), psoriasis (8.0% versus 6.9%, *P* = 0.03), and treatment with oral corticosteroids (14.0% versus 10.4% in 2009, *P* < 0.001).

**Conclusion:**

This nationwide, register-based study demonstrated a prevalence of clinically diagnosed ankylosing spondylitis of 0.18%. It revealed phenotypical and treatment differences between the sexes, as well as geographical and socio-economic differences in disease prevalence.

**Electronic supplementary material:**

The online version of this article (doi:10.1186/s13075-015-0627-0) contains supplementary material, which is available to authorized users.

## Introduction

Ankylosing spondylitis (AS) is a chronic inflammatory disorder primarily involving the sacroiliac joints and spine. It is associated with both articular and extra-articular clinical manifestations, including peripheral arthritis, enthesitis, anterior uveitis, psoriasis, and inflammatory bowel disease. The first symptoms usually occur before the age of 30 and seldom occur after the age of 45 [1]. The chronic and often progressive nature of the disease affects individuals for most of their working lives, limiting physical function, the ability to work, and perceived quality of life [2,3]. Pharmacological treatments include non-steroidal anti-inflammatory drugs (NSAIDs), oral glucocorticoids, synthetic disease modifying anti-rheumatic drugs (sDMARDs), and tumor necrosis factor inhibitors (TNFi) [4].

Compared with rheumatoid arthritis (RA), few studies have examined the prevalence of AS. The prevalence in Europe, North America, and China is estimated at 0.03 to 1.8% [5-17]; however, estimates are lower in Japan [18] and higher in populations with a high frequency of the major risk gene, HLA-B27 [19].

The highly varying estimates in the West may reflect differences in study methodology. Studies used different sampling sources, including local health records [8,13,10], small population surveys [12,11,5,7,6,9], general epidemiological surveys [14], blood donor registers [16], and regional patient registers [15]. The population surveys performed to date also used different screening methods, including telephone interviews [5], postal surveys [6,9], and home-based interviews [7].

Accurate and contemporary prevalence estimates, including the frequency of AS-related clinical manifestations and pharmacological treatments, as well as socio-economic and geographical variations, are important for healthcare planning, and they may provide clues to possible risk factors for the disease. Furthermore, little is known about differences in disease manifestations and pharmacological treatments between the sexes at the population level. One approach to obtaining such information is to use national healthcare registers, a method that has only been used on a regional level for AS [15], but was successfully used on a national level for RA [20]. This approach is supported by the high validity of the Swedish National Patient Register (NPR) in general [21], and by our recent assessment of the validity of the International Classification of Disease (ICD) codes [22] for AS in the Swedish NPR; the ICD codes showed high validity with regard to fulfilling the established classification criteria for both AS and spondyloarthritis (SpA) [23,24].

The primary aim of the present study was to assess the total national point prevalence of clinically diagnosed AS in Sweden in December 2009, and to stratify the prevalence according to age, sex, geographical and socio-economic factors. We also stratified the prevalence according to AS-related clinical manifestations and pharmacological treatments. The secondary aim was to compare disease manifestations and pharmacological treatments between the sexes.

## Methods

### Setting

The data used in this nationwide, population-based study were obtained from the Swedish national healthcare registers.

Healthcare provision in Sweden is largely funded by the taxpayer and is independent of individual financial or insurance considerations. There is an upper limit to an individual’s yearly costs for medical consultations and prescription medications. Patients with an inflammatory rheumatic disease such as AS are usually diagnosed at public or (less commonly) private rheumatology clinics. Such cases are rarely definitively diagnosed in a primary care setting [25].

Ethical approval for the study was granted by the Regional Ethics Committee, Karolinska Institute, Stockholm, Sweden. Patient consent was waived, as data were derived either from administrative registers that do not require informed consent or quality registers where the consent is already given at the time point of first registration.

### Data sources

The NPR comprises the Inpatient Register (IPR) and the Outpatient Register (OPR). The IPR was launched in 1964, and complete national coverage (almost 100%) was achieved in 1987. The IPR contains data (medical and administrative) derived from all patients discharged from hospitals in Sweden [21]. The OPR for public specialized care was started in 2001. The national coverage of the OPR is lower (approximately 73% in 2008 and 87% in 2011) [26,27] than that of the IPR, mainly due to missing data from private caregivers. Visits to primary care facilities are not covered by the NPR. Diagnoses are registered using the Swedish version of the ICD codes [22]. The present study used the NPR to identify AS cases and AS-related clinical manifestations between 1967 and 2009. An additional table shows the ICD codes used in the present study (Additional file 1: Table S1).

The Swedish Prescribed Drug Register [28] contains information about all prescribed drugs dispensed by Swedish pharmacies since July 2005. This register was used to determine the exposure of patients with a registered diagnosis of AS to NSAIDs, oral corticosteroids, and sDMARDs during 2009. Exposure to TNFi during 2009 was based on data retrieved from the Swedish Biologics Register (the Anti-Rheumatic Therapy in Sweden Register; ARTIS) [29], which was initiated in 1999 and includes over 85% of subcutaneous and intravenous TNFi prescribed for AS [30]. An individual was considered to be “exposed” in 2009 if they had at least one prescription dispensed or, in the case of TNFi, were listed in ARTIS as receiving medication during 2009.

Demographic data were obtained from Statistics Sweden [31], which holds data on immigration, emigration, and residency, as well as data on socio-economic factors (for example, marital status and level of formal education) for all those residing in Sweden.

Vital status on 31 December 2009 was determined via the cause-of-death register [32], which provides information on the date and cause(s) of death for all residents since 1961.

All data in the Swedish national healthcare registers are accessed via a personal identification number, which provides the link between all of the registers used in the present analyses [33].

### Definition of ankylosing spondylitis

**Figure 1:**
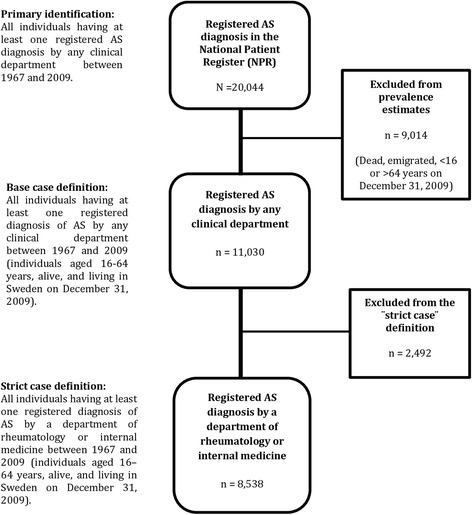
Flow chart showing the process of identifying patients with a registered ankylosing spondylitis (AS) diagnosis according to the World Health Organization International Classification of Disease codes in the National Patient Register between 1967 and 2009 according to the base case and strict case definition.

All individuals with an ICD diagnosis registration of AS between 1967 and 2009 were identified from the NPR (Figure 1). The base case definition required at least one AS diagnosis to be registered in the NPR (IPR 1967 to 2009 and/or OPR 2001 to 2009) by any department at a specialized care center. The strict case definition required at least one AS diagnosis to be registered by a department of rheumatology or internal medicine (Figure 1), a definition that we have previously found to have a high validity [23].

The case definitions used herein included individuals who had received their AS diagnosis for the first time before the age of 16, and also individuals diagnosed with juvenile idiopathic arthritis who had a subsequent diagnosis of AS. This is because these conditions were considered to be part of the same adult AS phenotype.

Results were obtained for individuals aged 16 to 64 years in 2009. The upper age limit was chosen to ensure a high case identification and at the same time minimize the flawing of the results which could be possibly caused by the inclusion of older individuals. Although >95% of patients with AS experience disease onset before the age of 45, there is a considerable delay (8 to 15 years) in obtaining a definitive diagnosis from a specialist in Sweden [23,15], meaning that a high age limit was required in order to maximize the number of cases enrolled. Moreover, since the major source used to identify AS cases, the OPR, did not start until 2001, the ability to accurately identify AS cases in older age groups with earlier disease onset, and who were exclusively diagnosed in a specialized outpatient care setting, was limited. Estimates of disease prevalence in older age groups may also be flawed due to the high mortality among AS patients [34]. Also, it may be that few AS diagnoses are made in older age groups due to either the limited impact of the disease on older people [35] or the influence of other major age-related co-morbidities on diagnosis registration patterns.

### Geographical variations and level of formal education

There are six healthcare regions in Sweden: one northern, one southern, and four in between (the southeastern and western regions, and the Uppsala-Örebro and Stockholm regions). These regions were used to calculate the regional prevalence of clinically diagnosed AS and are presented in Additional file 2: Figure S1.

The level of formal education was classified into three categories (≤9 years, 10 to 12 years, and >12 years of schooling). The prevalence of clinically diagnosed AS in relation to the level of education is presented for those aged ≥30 years, the age by which the majority of the population is expected to have completed their studies.

### Statistical analysis

The total point prevalence of clinically diagnosed AS was estimated on 31 December 2009 according to both the base case and strict case definitions, and after stratification according to sex and age. Despite the cumulative ascertainment of AS cases, the term “point prevalence” is used. Considering that AS is a chronic disease, individuals with a previous registered AS diagnosis, alive and living in Sweden in 2009, are assumed having an AS diagnosis on 31 December 2009 as well. The point prevalence of AS was thus calculated by taking the number of AS individuals according to each definition and dividing it by the Swedish population on 31 December 2009 with similar age. Furthermore, the prevalence was estimated for subgroups based on AS-related clinical manifestations and pharmacological treatment, by taking the number of AS individuals (according to the base case definition) having dispensed prescriptions for each pharmacological treatment during any part of 2009, or those with a registered diagnosis of AS-related disease manifestations between 1967 and 2009, and dividing it by the Swedish population on 31 December 2009 with similar age. Crude and age-/sex-standardized point prevalence estimates according to healthcare region and level of formal education were also calculated. Standardization was performed to the Swedish population aged 16 to 64 years in 2009. For comparisons between the sexes, the Chi-squared test was used for categorical variables, and the Student’s *t*-test was used for normally distributed continuous variables. *P*-values <0.05 were considered statistically significant. For comparisons between geographical location and socio-economic factors, statistical significance was based on the absence of overlap between the estimated 95% confidence interval (CI). Logistic regression models adjusted for age and AS-related clinical manifestations (psoriasis, inflammatory bowel disease, anterior uveitis, and peripheral arthritis) were used to examine sex-related differences in treatment patterns. All analyses were performed using IBM SPSS Statistics for Windows, Version 20.0. Armonk, NY: IBM Corp and Microsoft Excel 2010, Microsoft Corporation, One Microsoft Way, Redmond, WA, USA.

## Results

On 31 December 2009, the population of Sweden was about 9.3 million, of which 64% (5,982,237) were aged 16 to 64 years (the denominator in this study). Males accounted for 50.8%.

We identified 20,044 AS cases registered in the NPR between 1967 and 2009 (Figure 1). Of those, 11,030 were aged between 16 and 64 years, alive, and living in Sweden on 31 December 2009. Of these 11,030 individuals, 76% (n = 8,379) were registered in the OPR, 48.5% (n = 5,350) in the IPR, and 24.5% (n = 2,699) in both. Of those fulfilling the base case definition of AS, 7,041 (63.8%) were male. The mean age in 2009 was 48.3 years (standard deviation (SD) 11.3) and the mean age at the time of first AS diagnosis registration in the NPR was 38.6 years (SD 11.4) (Table 1). Of those fulfilling the base case definition, 77.4% (n = 8,538) also fulfilled the strict case definition (Figure 1).

**Table 1:** **Demographics, pharmacological treatment, and ankylosing spondylitis (AS)-related clinical manifestations in patients with an ankylosing spondylitis diagnosis in the National Patient Register (1967 to 2009) according to the base case and strict case definitions**

	**Base case definition**				**Strict case definition**
	**Men**	**Women**		**Total**	**Total**
	**(N = 7,041)**	**(N = 3,989)**		**(N = 11,030)**	**(N = 8,538)**
**Demographics**	**Mean (SD)**		***P*** **-value**	**Mean (SD)**	**Mean (SD)**
Age at diagnosis registration	38.6 (11.4)	38.6 (11.3)	0.907	38.6 (11.4)	39.1 (11.0)
Age on 31 December 2009	48.6 (11.2)	47.6 (11.5)	<0.001	48.3 (11.3)	48.5 (10.9)
**Pharmacological treatment during 2009** ^**†**^	**N (% of total)**		***P*** **-value** ^**†††**^	**N (% of total)**	
NSAIDs	3,724 (52.9)	2,164 (54.2)	0.169	5,888 (53.4)	4,736 (55.5)
Oral corticosteroids	734 (10.4)	557 (14.0)	<0.001	1,291 (11.7)	1,078 (12.6)
sDMARDs	1,406 (20.0)	841 (21.1)	0.163	2,247 (20.4)	1,937 (22.7)
TNFi	1,100 (15.6)	471 (11.8)	<0.001	1,571 (14.2)	1,442 (16.9)
Any pharmacological treatment	4,634 (65.8)	2,653 (66.5)	0.460	7,287 (66.1)	5,974 (70.0)
**AS-related clinical manifestations (1967–2009)** ^**††**^	**N (% of total)**		***P*** **-value**	**N (% of total)**	
Anterior uveitis	1,797 (25.5)	797 (20.0)	<0.001	2,594 (23.5)	2,109 (24.7)
Psoriasis	486 (6.9)	320 (8.0)	0.030	806 (7.3)	669 (7.8)
IBD	571 (8.1)	343 (8.6)	0.371	914 (8.3)	746 (8.7)
Peripheral arthritis	1,079 (15.3)	864 (21.7)	<0.001	1,943 (17.6)	1,568 (18.4)
Hip arthroplasty	527 (7.5)	319 (8.0)	0.331	846 (7.7)	658 (7.7)

Synthetic disease modifying anti-rheumatic drugs (sDMARDs): methotrexate, sulfasalazine, and leflunomide (according to Anatomical Therapeutic Chemical (ATC) codes in the prescribed drugs register). Any pharmacological treatment: non-steroidal anti-inflammatory drugs (NSAIDs), oral corticosteroids, or sDMARDs (according to ATC codes in the prescribed drugs register), or tumor necrosis factor inhibitors (TNFi) (according to the Anti-Rheumatic Therapy in Sweden Register (ARTIS)). IBD: inflammatory bowel disease (according to International Classification of Disease (ICD) codes in the National Patient Register (NPR)). Peripheral arthritis: psoriatic arthritis, reactive arthritis, polyarthritis, or any type of arthritis (according to ICD codes in the NPR). ^†^At least one dispensed prescription during 2009 according to the Swedish prescribed drugs register or listed in ARTIS as receiving TNF-α medication during 2009. ^††^At least one diagnosis registered in the NPR between 1967 and 2009. ^†††^Adjusted for age and a registered diagnosis of anterior uveitis, psoriasis, IBD, or peripheral arthritis. SD, standard deviation.

### Prevalence of clinically diagnosed ankylosing spondylitis, pharmacological treatment, and disease manifestations

**Figure 2:**
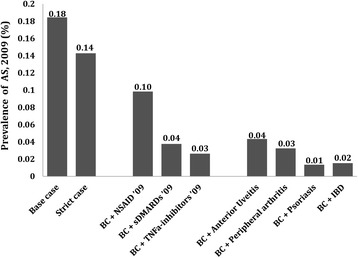
The point prevalence of clinically diagnosed ankylosing spondylitis (AS) in Sweden on 31 December 2009 among those aged 16 to 64 years according to the base case and strict case definitions, and the prevalence of clinically diagnosed AS in 2009 for subgroups stratified according to pharmacological treatment and disease-related clinical manifestations. Peripheral arthritis defined as psoriatic arthritis, reactive arthritis, polyarthritis, or any type of arthritis. BC, base case; IBD, inflammatory bowel disease; NSAID, non-steroidal anti-inflammatory drug; sDMARD, synthetic disease modifying anti-rheumatic drug; TNF, tumor necrosis factor.

The total point prevalence of clinically diagnosed AS (16 to 64 years old) on 31 December 2009, according to both base case and strict case definitions, was 0.18% (11,030/5,982,237) and 0.14% (8,538/5,982,237), respectively. Of the AS cases identified between 1967 and 2009 according to the base case definition, 68.4% had a registered visit in the NPR during the period 2005 to 2009 (7,550/11,030). The prevalence of AS according to the base case definition having any dispensed prescription for NSAIDs during 2009 was 0.10%, while the prevalence for those with any dispensed prescription for sDMARDs or TNFi during 2009, or those registered with AS-related clinical manifestations between 1967 and 2009, was considerably lower (Figure 2).

There were differences between the sexes with regard to pharmacological treatments received during 2009. Women were more often treated with oral corticosteroids than men (*P* < 0.001), whereas men were more often treated with TNFi (*P* < 0.001) (Table 1). There were also differences between the sexes regarding some AS-related clinical manifestations. Anterior uveitis was more common in men (*P* < 0.001), and peripheral arthritis and psoriasis were more common in women (*P* < 0.001 and *P* = 0.03, respectively) (Table 1). The sex-related differences with regard to treatment with TNFi (odds ratio = 1.48; 95% CI, 1.32 to 1.67; *P* < 0.001) and oral corticosteroids (odds ratio = 1.35; 95% CI, 1.20 to 1.52; *P* < 0.001) remained significant after adjustment for age and AS-related clinical manifestations (psoriasis, inflammatory bowel disease, anterior uveitis, and peripheral arthritis) in logistic regression models.

Data regarding demographics, disease manifestations, pharmacological treatment, and sex-related differences were similar regardless of whether the base case or strict case definitions of AS were used, as shown in detail in Additional file 3: Table S2.

**Figure 3:**
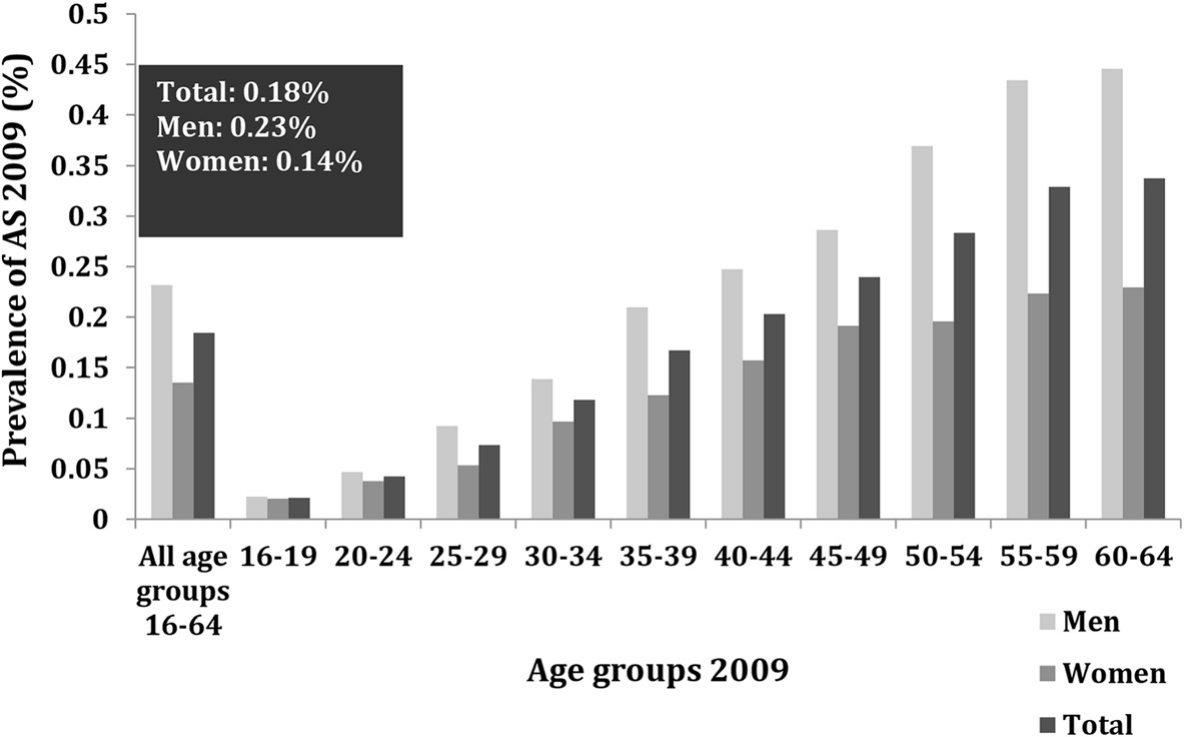
The age- and sex-stratified point prevalence of clinically diagnosed ankylosing spondylitis (AS) in Sweden on 31 December 2009, according to the base case definition.

The prevalence of clinically diagnosed AS increased linearly with age up to the age of 55 years, and then reached a plateau. For all age groups combined, the prevalence of AS was higher in men than in women (0.23% versus 0.14%; *P* < 0.001). The male-to-female ratio was highest after the age of 50; however, it was lower for younger age groups (Figure 3). The same pattern was observed for the prevalence of AS according to the strict case definition after age and sex stratification (Additional file 4: Figure S2).

### Geographical location

**Figure 4:**
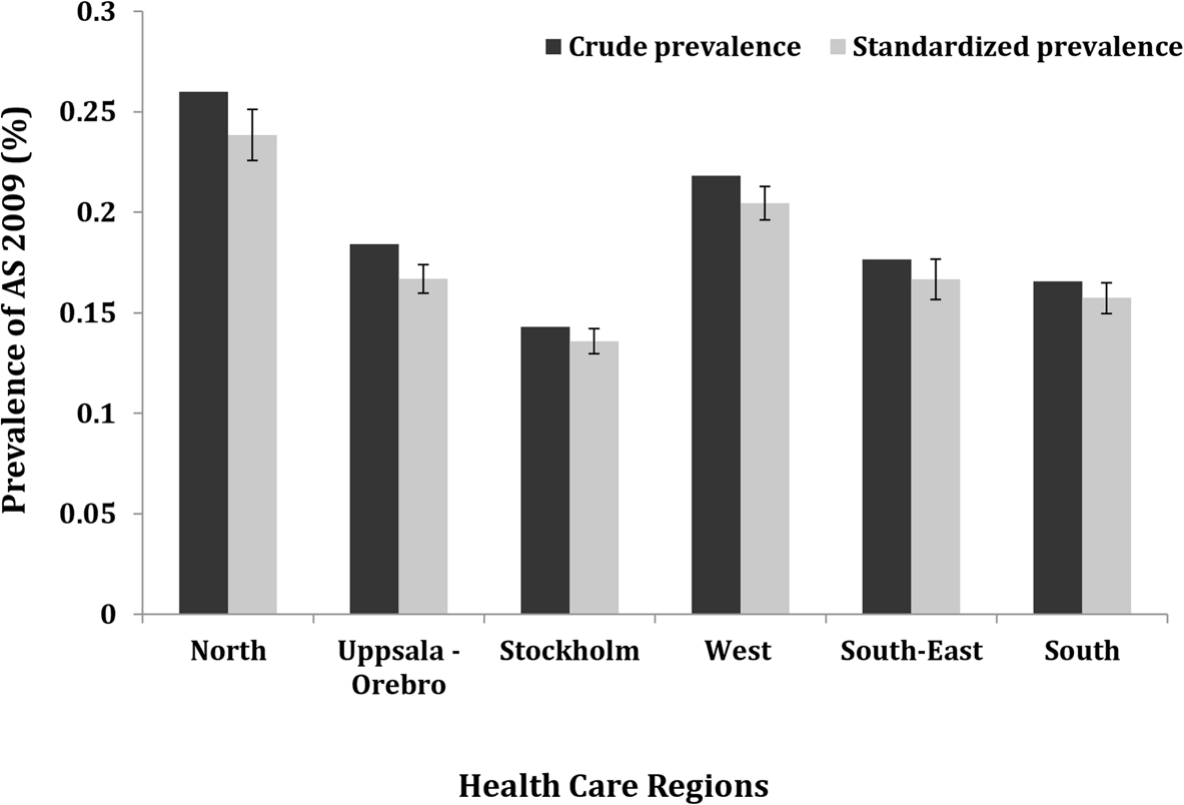
The point prevalence of clinically diagnosed ankylosing spondylitis (AS) in Sweden on 31 December 2009 according to the base case definition, among those aged 16 to 64 years in each healthcare region (crude and standardized according to age and sex).

Estimates according to healthcare region revealed that the age-/sex-standardized prevalence of clinically diagnosed AS was highest in the Northern Healthcare Region, which was 0.24% (95% CI, 0.23 to 0.25%), whereas that in the Southern Region was 0.16% (95% CI, 0.15 to 0.16%); however, there was no clear north–south gradient with regard to prevalence of AS across the rest of the country (Figure 4).

### Level of formal education

**Figure 5:**
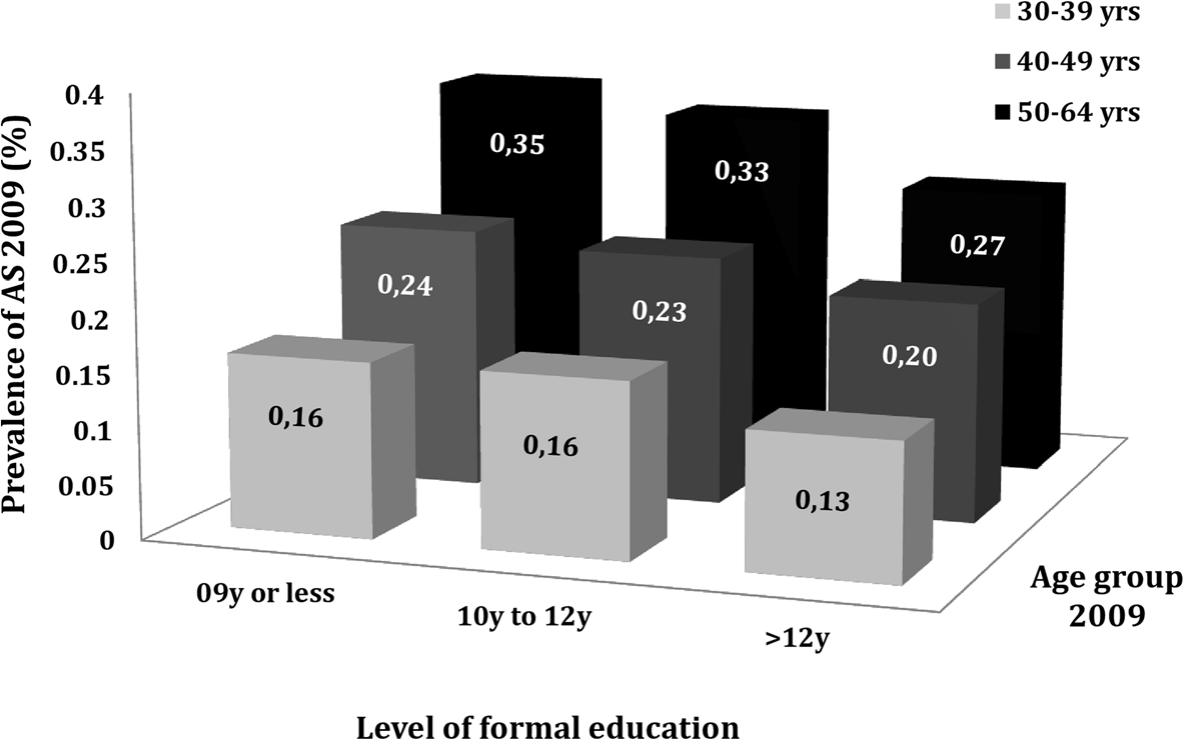
The point prevalence of clinically diagnosed ankylosing spondylitis (AS) in Sweden on 31 December 2009 (according to the base case definition), among those aged 30 to 64 years, stratified according to the level of formal education and age in 2009.

The age-/sex-standardized prevalence of clinically diagnosed AS was lowest in the population group with >12 years of formal education (0.20%; 95% CI, 0.19 to 0.21%). A higher prevalence was observed in those with a lower level of education: ≤9 years (0.24%; 95% CI, 0.22 to 0.25%) or 10 to 12 years (0.24%; 95% CI, 0.23 to 0.24%) of schooling, as shown in Additional file 5: Figure S3. A lower prevalence of AS in the population with the highest level of education was still observed after stratification according to age in 2009 (Figure 5).

## Discussion

This nationwide, register-based study revealed that the point prevalence of clinically diagnosed AS in Sweden in 2009 was 0.18%. The prevalence was lower in those with the highest level of education and higher in those living in the north. Furthermore, the prevalence was higher in men than women. In addition, the frequency of anterior uveitis and treatment with TNFi was higher in men than in women. On the other hand, women had more registered diagnoses related to peripheral arthritis and psoriasis, and were more frequently treated with oral corticosteroids.

Estimates of AS prevalence vary considerably. Differences in study design and population genetics are the most likely explanations; however, geographical, environmental, and socio-economic factors may also play a role. In northern Norway, the prevalence of AS is reported to be 0.21% [13], which is very similar to our own estimates for northern Sweden. A population survey from Finland [12], which examined a representative sample of the population aged ≥30 years (The Mini-Finland Health Survey) reported a prevalence of 0.15%. Finally, a regional register-based study from southern Sweden [15] estimated the prevalence of AS in those ≥15 years old to be 0.12%, which is also consistent with the results we obtained for the southern region of Sweden when using the same age restriction and case definition criteria (data not shown).

According to the results presented herein, sex appears to play a role in AS prevalence. First, the prevalence of AS was higher in men than women across all age groups; this was expected given the results of previous studies [12,15,7]. Furthermore, a registered visit with a diagnosis of anterior uveitis was more common among men. Men were also more likely to be prescribed TNFi than women. By contrast, a diagnosis indicating peripheral arthritis and psoriasis, and a prescription for oral corticosteroids were more common among women. These findings support the presence of sex-related phenotypic differences, which could affect treatment choices. However, the differences between the sexes with regard to pharmacological treatment remained significant, even after adjusting for such phenotypic differences.

We also found geographical and socio-economic differences in AS prevalence. Previous studies may have lacked sufficient data or the power to evaluate such differences [5,7]. The higher prevalence in northern Sweden (identified herein) and in northern Scandinavia [11,13,14] may be explained by population genetics; the prevalence of the AS-related genotype, HLA-B27, is high (24%) in the indigenous population (Sami) in these areas [11]. The frequency of HLA-B27 is lower in the non-Sami populations of northern Scandinavia (10 to 16%) and Finland (12 to 16%); however, it is still considerably higher than that in southern Europe [19]. Additionally, the present study is the first to suggest an inverse relationship between AS prevalence and the level of education. Such a relationship was also shown previously for RA [20]. It may illustrate that the (usually) early age of AS onset may affect one’s ability to pursue education; however, it may also indicate that socio-economic or environmental factors may play a role in AS development. The fact that those exclusively followed in private care are not included in our study may introduce a selection bias that affects this analysis. Those with higher educational attainment (indicating higher socio-economic status) may be more likely to be treated in private clinics, and therefore are missing from our sample. However, in Sweden the majority of private healthcare providers are reimbursed by the counties for providing services covered by the same regulations and fees that apply to municipal care facilities, suggesting that the ability to contact a private rheumatologist is not strongly dependent on socio-economic status. Future studies could focus on identifying environmental factors associated with AS by linking the populations examined in the present study to other registers containing information regarding relevant early life exposure.

The present study has several strengths. First, it is the first nationwide prevalence study on clinically diagnosed AS. Previous studies were either based on regional or small national population samples, with limited generalizability. Second, in contrast to smaller regional studies, we used a uniform case ascertainment over a large geographical area, enabling us to demonstrate geographical variations in disease occurrence, which may reflect differences in genetic, environmental, and socio-economic factors. Third, the register-based approach (using AS diagnoses made by physicians) reflects the real disease burden as estimates of AS occurrence are based on clinical diagnoses, and enables the assessment of the frequency of AS-related clinical manifestations and pharmacological treatments.

The study also has a number of limitations. First, a register-based approach does not identify patients not seeking contact with a healthcare practitioner, since it only identified clinically diagnosed cases. Second, the NPR does not cover AS cases that are managed exclusively in a primary care or private setting. According to a previous study from southern Sweden, only 3% of AS patients are being followed exclusively in primary care [25], usually those with a mild disease course. In another study, the proportion of AS cases solely seeking private healthcare was estimated to be 15% [15]. Our own estimates from the biologics register show that only 4.6% of those receiving TNFi are treated exclusively privately (data not shown). Taking these factors into consideration, although we acknowledge that in particular some AS cases with mild disease or short disease duration may not be included in the present study, our underestimation of AS prevalence should be less than 10%. Finally, the validity of an AS diagnosis registered in the NPR may be questioned, as all diagnoses are based on the judgment of each individual physician and not on established classification criteria. However, we previously showed that AS diagnoses according to the strict case definition have high validity, with a positive-predictive value of 80% for fulfilling the modified New York criteria, and 89% for fulfilling any of the commonly used criteria for SpA [23]. These findings indicate that only a low percentage of patients with clinical non-radiographic axial SpA are incorrectly classified as AS in the NPR. Based on these findings, misclassification of AS diagnosis in the NPR is unlikely to have a significant impact on our results. Finally, the present study specifically addresses the prevalence of clinically diagnosed AS and not that for the whole group of axial SpA (including the non-radiographic stage). The latter is expected to be higher and thus more studies are necessary to estimate the total prevalence of axial SpA.

## Conclusions

To conclude, this nationwide, register-based prevalence estimate of clinically diagnosed AS in Sweden offers a unique opportunity to improve our understanding of the real burden that AS places on the healthcare system. We identified phenotypical and pharmacological treatment differences between the sexes, and differences in disease prevalence related to geographical region and socio-economic factors, suggesting that genetic and (possibly) environmental factors may be important for the development of AS.

## Additional files

Additional file 1: Table S1. Diagnostic codes indicating a diagnosis of ankylosing spondylitis (AS) and AS-related clinical manifestations according to three consecutive Swedish versions of the World Health Organization International Classification of Disease codes. Additional file 2: Figure S1. The Swedish Healthcare regions. Additional file 3: Table S2. Demographics, pharmacological treatments, and ankylosing spondylitis (AS)-related clinical manifestations in patients with an AS diagnosis registered in the National Patient Register (NPR) (1967 to 2009) according to the strict case definition. Additional file 4: Figure S2. Age- and sex-stratified point prevalence of clinically diagnosed ankylosing spondylitis (AS) in Sweden on 31 December 2009, according to the strict case definition. Additional file 5: Figure S3. The point prevalence of clinically diagnosed ankylosing spondylitis (AS) in Sweden on 31 December 2009 (according to the base case definition), among those aged 30 to 64 years, stratified according to the level of formal education (both crude and standardized according to age and sex).

## Abbreviations

ARTIS: Anti-Rheumatic Therapy in Sweden Register
AS: ankylosing spondylitis
CI: confidence interval
ICD: International Classification of Disease
IPR: Inpatient Register
NPR: National Patient Register
NSAID: non-steroidal anti-inflammatory drug
OPR: Outpatient Register
RA: rheumatoid arthritis
SD: standard deviation
sDMARD: synthetic disease modifying anti-rheumatic drug
SpA: spondyloarthritis
TNFi: tumor necrosis factor inhibitors
