# Supplementary material for: The prevalence of clinically diagnosed ankylosing spondylitis and its clinical manifestations: a nationwide register study
Source: Arthritis Res Ther. 2015 May 9;17(1):118. doi: 10.1186/s13075-015-0627-0 (PMC4424886; doi:10.1186/s13075-015-0627-0)
Supplement: Additional file 1: Table S1. — Diagnostic codes indicating a diagnosis of ankylosing spondylitis (AS) and AS-related clinical manifestations according to three consecutive Swedish versions of the World Health Organization International Classification of Disease codes. [file 13075_2015_627_MOESM1_ESM.pdf]

Additional table 1. Diagnostic codes indicating a diagnosis of AS and AS-related clinical manifestations according to three consecutive Swedish versions of the WHO International Classification of Disease codes

	S-ICD8	S-ICD9	S-ICD-10
**Ankylosing spondylitis**	712.40, 726.99	720A	M45
**Anterior uveitis**		364A–364B	H20, H221
**Psoriasis**	696	696	L40
**Inflammatory bowel disease**	563.00, 563.10, 569.02	555–556	K50–K51
**Peripheral arthritis***			
Reactive arthritis		711B, 711D–711W	M013–M029, M036
Psoriatic arthritis	696.00	696A, 713D	L405, M070, M071, M072, M073
Polyarthritis	715.99	714X, 716F	M130
Other arthritis		716F–716X, 714X	M13
**Hip arthroplasty**			NFB, 8,400–8,415, 8,419

**Any of the below*
