# Supplementary figures and images for: The prevalence of clinically diagnosed ankylosing spondylitis and its clinical manifestations: a nationwide register study
Source: Arthritis Res Ther. 2015 May 9;17(1):118. doi: 10.1186/s13075-015-0627-0 (PMC4424886; doi:10.1186/s13075-015-0627-0)

**Additional figure 1.** The Swedish Healthcare Regions.

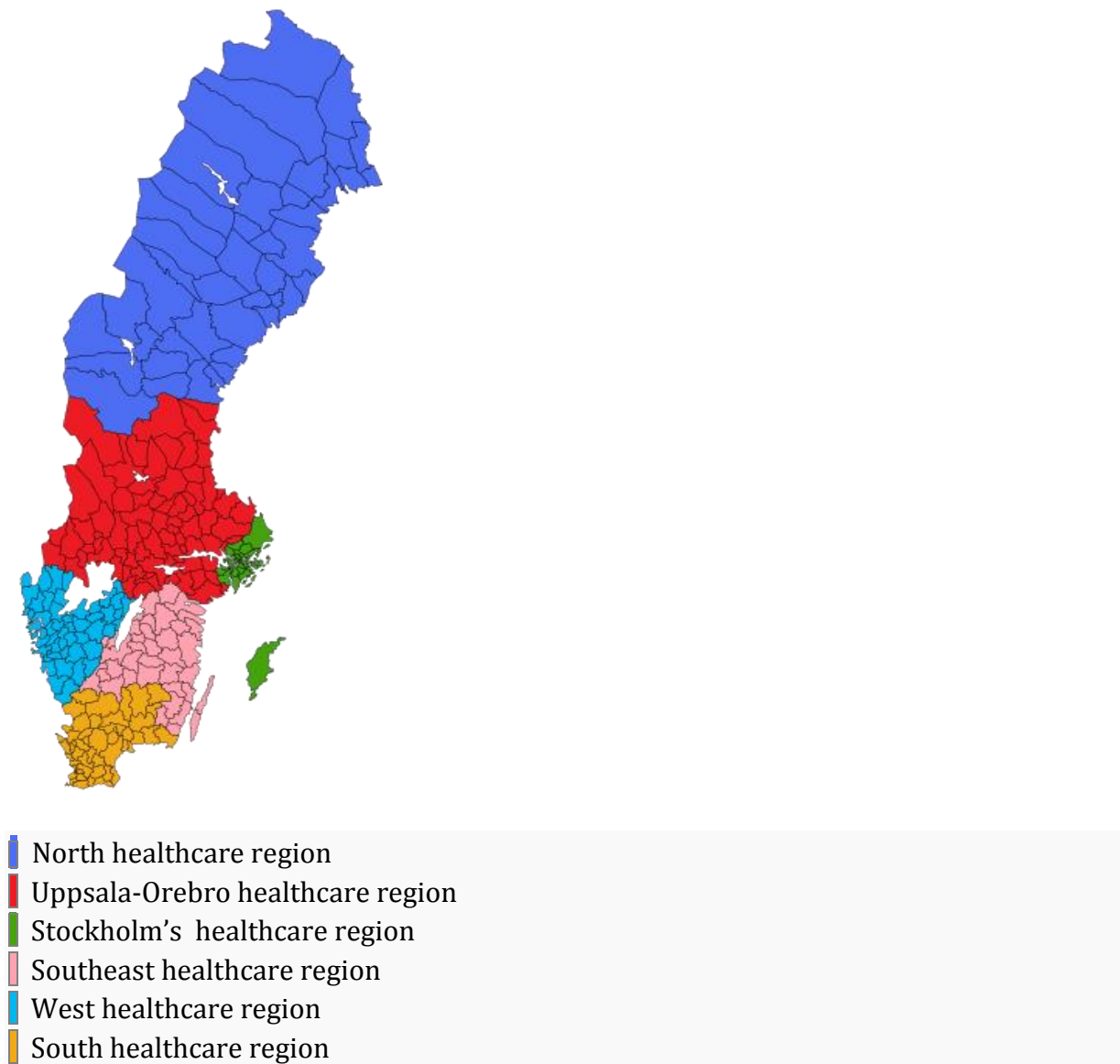

Supplement: Additional file 2: Figure S1. — The Swedish Healthcare regions. [file 13075_2015_627_MOESM2_ESM.pdf]
