# Supplementary material for: The prevalence of clinically diagnosed ankylosing spondylitis and its clinical manifestations: a nationwide register study
Source: Arthritis Res Ther. 2015 May 9;17(1):118. doi: 10.1186/s13075-015-0627-0 (PMC4424886; doi:10.1186/s13075-015-0627-0)
Supplement: Additional file 3: Table S2. — Demographics, pharmacological treatments, and ankylosing spondylitis (AS)-related clinical manifestations in patients with an AS diagnosis registered in the National Patient Register (NPR) (1967 to 2009) according to the strict case definition. [file 13075_2015_627_MOESM3_ESM.pdf]

Additional table 2. Demographics, pharmacological treatments, and AS-related clinical manifestations in patients with an AS diagnosis registered in the National Patient Register (NPR) (1967–2009) according to the strict case definition.

	Strict case definition			
	**Men (N = 5,580)**	**Women (N = 2,958)**		**Total (N = 8,538)**
**Demographics**	**Mean (SD)**		**P-value**	**Mean (SD)**
Age at diagnosis registration	38.3 (11.0)	38.7 (10.8)	0.087	39.1 (11.0)
Age on December 31, 2009	48.7 (10.9)	48.1 (10.9)	0.028	48.5 (10.9)
**Pharmacological treatment during 2009†**	**N (% of total)**		**P-value†††**	**N (% of total)**
NSAIDs	3,065 (54.9)	1,671 (56.5)	0.167	4,736 (55.5)
Oral corticosteroids	634 (11.4)	444 (15.0)	<0.001	1,078 (12.6)
sDMARDs	1,242 (22.3)	695 (23.5)	0.194	1,937 (22.7)
TNFi	1,035 (18.5)	407 (13.8)	<0.001	1,442 (16.9)
Any pharmacological treatment	3,895 (69.8)	2,079 (70.3)	0.644	5,974 (70.0)
**AS-related clinical manifestations (1967-2009) ††**	**N (% of total)**		**P-value**	**N (% of total)**
Anterior uveitis	1,477 (26.5)	632 (21.4)	<0.001	2,109 (24.7)
Psoriasis	411 (7.4)	258 (8.7)	0.026	669 (7.8)
IBD	484 (8.7)	262 (8.9)	0.775	746 (8.7)
Peripheral arthritis	890 (15.9)	678 (22.9)	<0.001	1,568 (18.4)
Hip arthroplasty	418 (7.5)	240 (8.1)	0.305	658 (7.7)

sDMARDs: methotrexate, sulfasalazine, and leflunomide (according to ATC codes in the prescribed drugs register); **Any medicine:** NSAIDs, oral corticosteroids, sDMARDs (according to ATC codes in the prescribed drugs register), or TNFi (according to ARTIS); **IBD:** inflammatory bowel disease (according to ICD-codes in the NPR); **Peripheral arthritis:** Psoriatic arthritis, reactive arthritis, polyarthritis, or any type of arthritis (according to ICD-codes in the NPR); **NSAIDs:** Non-steroidal anti-inflammatory drugs; **sDMARDs:** synthetic disease modifying anti-rheumatic drugs; **TNFi:** tumor necrosis factor alpha inhibitors.

*Data regarding dispensed pharmacologic treatments were obtained from the Swedish prescribed drugs register during 2009. Data regarding TNFi were extracted from the national Swedish Biologics Register (ARTIS) during 2009.

†At least one dispensed prescription during 2009 according to the Swedish prescribed drugs register or listed in ARTIS as receiving TNFi medication during 2009.

††At least one diagnosis registered in the NPR between 1967 and 2009.

†††Adjusted for age and a registered diagnosis of anterior uveitis, psoriasis, IBD, or peripheral arthritis.
