# Supplementary material for: The prevalence of clinically diagnosed ankylosing spondylitis and its clinical manifestations: a nationwide register study
Source: Arthritis Res Ther. 2015 May 9;17(1):118. doi: 10.1186/s13075-015-0627-0 (PMC4424886; doi:10.1186/s13075-015-0627-0)

Additional figure 2. Age- and sex-stratified point prevalence of clinically diagnosed AS in Sweden on December 31, 2009, according to the strict case definition.

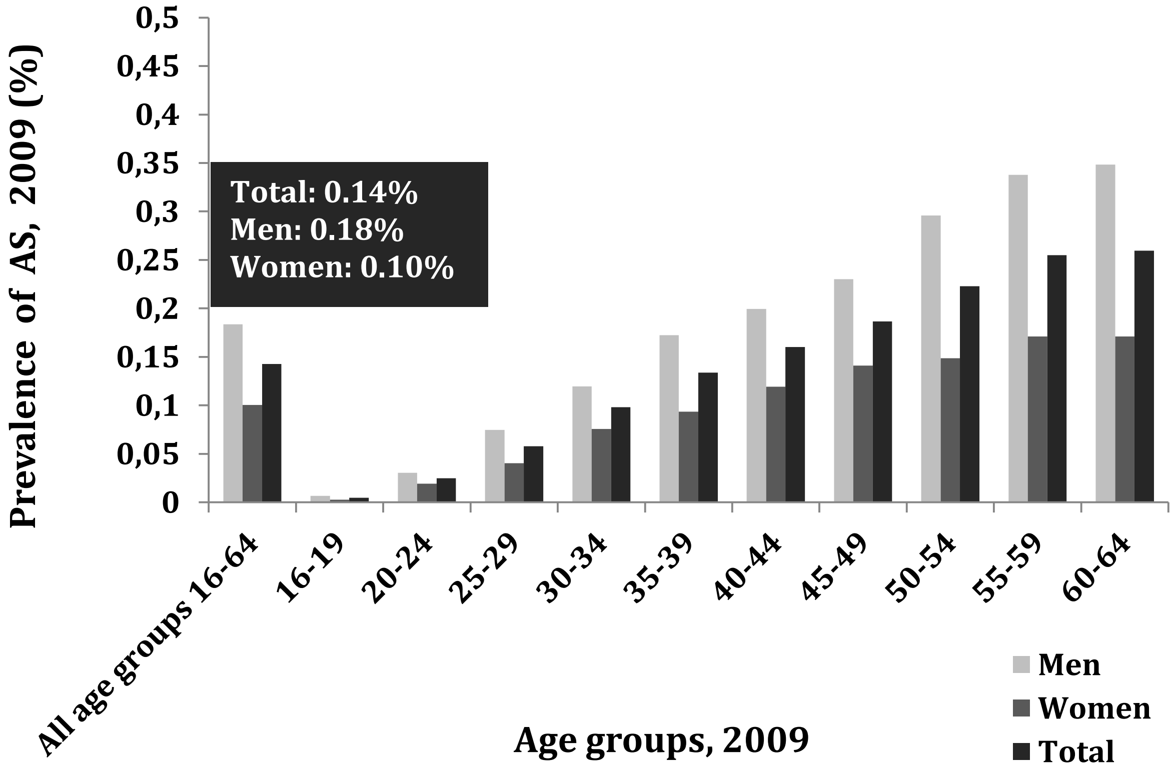

Supplement: Additional file 4: Figure S2. — Age- and sex-stratified point prevalence of clinically diagnosed ankylosing spondylitis (AS) in Sweden on 31 December 2009, according to the strict case definition. [file 13075_2015_627_MOESM4_ESM.pdf]
