# Supplementary material for: The prevalence of clinically diagnosed ankylosing spondylitis and its clinical manifestations: a nationwide register study
Source: Arthritis Res Ther. 2015 May 9;17(1):118. doi: 10.1186/s13075-015-0627-0 (PMC4424886; doi:10.1186/s13075-015-0627-0)

Additional figure 3. The point prevalence of clinically diagnosed AS in Sweden on December 31, 2009 (according to the base case definition), among those aged 30–64 years, stratified according to the level of formal education (both crude and standardized according to age and sex).

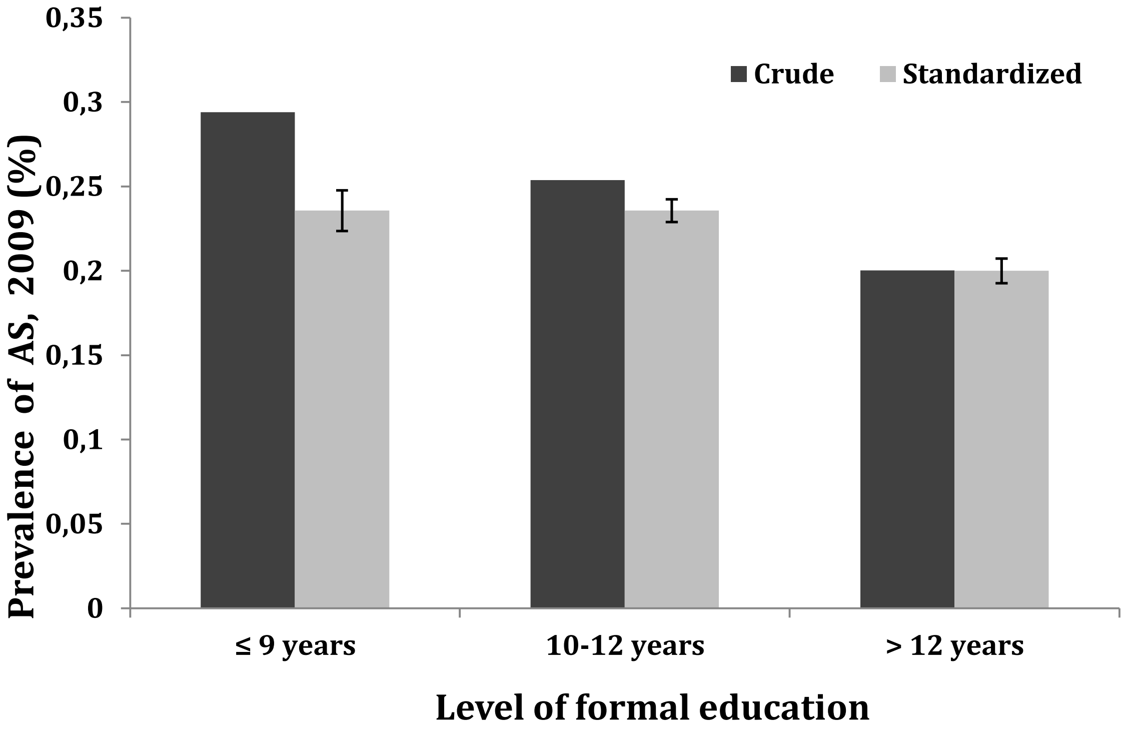

Supplement: Additional file 5: Figure S3. — The point prevalence of clinically diagnosed ankylosing spondylitis (AS) in Sweden on 31 December 2009 (according to the base case definition), among those aged 30 to 64 years, stratified according to the level of formal education (both crude and standardized according to age and sex). [file 13075_2015_627_MOESM5_ESM.pdf]
